# Characterization of Mucus-Related Properties of *Streptococcus thermophilus:* From Adhesion to Induction

**DOI:** 10.3389/fphys.2018.00980

**Published:** 2018-07-24

**Authors:** Neïké Fernandez, Laura Wrzosek, Joanna M. Radziwill-Bienkowska, Belinda Ringot-Destrez, Marie-Pierre Duviau, Marie-Louise Noordine, Valérie Laroute, Véronique Robert, Claire Cherbuy, Marie-Line Daveran-Mingot, Muriel Cocaign-Bousquet, Renaud Léonard, Catherine Robbe-Masselot, Françoise Rul, Eric Ogier-Denis, Muriel Thomas, Muriel Mercier-Bonin

**Affiliations:** ^1^Micalis Institute, INRA, AgroParisTech, Université Paris-Saclay, Jouy-en-Josas, France; ^2^Institute of Biochemistry and Biophysics, Polish Academy of Sciences, Warsaw, Poland; ^3^Université de Lille, Lille, France; ^4^USTL, UGSF, IFR 147, CNRS, Villeneuve-d’Ascq, France; ^5^UMR 8576, Unité de Glycobiologie Structurale et Fonctionnelle, Villeneuve-d’Ascq, France; ^6^LISBP, CNRS, INRA, INSA, Université de Toulouse, Toulouse, France; ^7^INSERM, Research Centre of Inflammation BP 416, Paris, France; ^8^University Paris Diderot, Sorbonne Paris Cité, Paris, France; ^9^Laboratory of Excellence Labex INFLAMEX, Université Sorbonne Paris Cité, Paris, France

**Keywords:** mucus, mucin, microbiota, gut, lactic acid bacteria, lactate, gnotobiotic rodent

## Abstract

Mucus is a major component of the intestinal barrier involved both in the protection of the host and the fitness of commensals of the gut. *Streptococcus thermophilus* is consumed world-wide in fermented dairy products and is also recognized as a probiotic, as its consumption is associated with improved lactose digestion. We determined the overall effect of *S. thermophilus* on the mucus by evaluating its ability to adhere, degrade, modify, or induce the production of mucus and/or mucins. Adhesion was analyzed *in vitro* using two types of mucins (from pig or human biopsies) and mucus-producing intestinal HT29-MTX cells. The induction of mucus was characterized in two different rodent models, in which *S. thermophilus* is the unique bacterial species in the digestive tract or transited as a sub-dominant bacterium through a complex microbiota. *S. thermophilus* LMD-9 and LMG18311 strains did not grow in sugars used to form mucins as the sole carbon source and displayed weak binding to mucus/mucins relative to the highly adhesive TIL448 *Lactococcus lactis*. The presence of *S. thermophilus* as the unique bacteria in the digestive tract of gnotobiotic rats led to accumulation of lactate and increased the number of Alcian-Blue positive goblet cells and the amount of the mucus-inducer KLF4 transcription factor. Lactate significantly increased KLF4 protein levels in HT29-MTX cells. Introduction of *S. thermophilus* *via* transit as a sub-dominant bacterium (10^3^ CFU/g feces) in a complex endogenous microbiota resulted in a slight increase in lactate levels in the digestive tract, no induction of overall mucus production, and moderate induction of sulfated mucin production. We thus show that although *S. thermophilus* is a poor mucus-adhesive bacterium, it can promote mucus pathway at least in part by producing lactate in the digestive tract.

## Introduction

*Streptococcus thermophilus* is a lactic acid bacterium traditionally used by the food industry because of its ability to ferment and acidify milk. It is one of the two bacteria used in yogurt, defined as “the coagulated milk product obtained by lactic acid fermentation through the action of *Lactobacillus delbrueckii* subsp. *bulgaricus* (*L. bulgaricus*) and *S. thermophilus* from milk and milk products" (FAO/WHO, 2002). *S. thermophilus* and *L. bulgaricus* are alive and abundant (at least 10^7^CFU/g) in the final product, with *S. thermophilus* being more abundant (Herve-Jimenez et al., 2009; Ben-Yahia et al., 2012). *S. thermophilus* is used as a dairy starter because it efficiently converts lactose into lactic acid, which rapidly reduces the pH and coagulates the caseins of milk. This bacterium also confers texture and taste properties to yogurt, such as viscosity, acidity, and water holding capacity (Rul, 2017). Aside from its technological advantages, *S. thermophilus* can provide health benefits to consumers. The presence of living bacteria in yogurt is associated with a better capacity to digest lactose for individuals with lactose intolerance. This is due to their β-galactosidase activity, which compensates the lactose-degrading deficiency of host intestinal cells. The health benefit conferred by the consumption of yogurt is sufficiently supported by scientific evidence that the claimed effect of aiding lactose digestion associated with live yogurt cultures has been acknowledged by the European Food Safety Authority [Efsa Panel on Dietetic Products, Nutrition and Allergies (NDA), 2010]. To date, the beneficial physiological effect provided by the two yogurt bacteria is the unique health claim related to the presence of living cultures. It may be worthwhile to explore other beneficial properties of *S. thermophilus* that could enlarge the health benefits linked to its consumption.

By degrading lactose, *S. thermophilus* produces lactate that can shape and reinforce endogenous bacterial communities of the intestinal microbiota in the digestive tract (Veiga et al., 2010). Lactate is a powerful antimicrobial factor that inhibits the growth of pathogens and participates in the trophic chain between microbial communities, because it favors the growth of bacteria that consume lactate and subsequently generate secondary short chain fatty acids, such as propionate and butyrate (Louis and Flint, 2017). Lactate is thus an intermediate metabolite used as a substrate by commensals. It may also be involved in the physiology of epithelial cells, although it is yet to be determined whether lactate, in the intestine is preferentially consumed by bacteria or host cells. The role of lactate as a mediator in the dialog between *S. thermophilus* and the host has been highlighted in an experimental rat model mono-colonized with the strain LMD-9 (Rul et al., 2011). The presence of LMD-9 alone in the digestive tract of these gnotobiotic rats results in the accumulation of high levels of lactate (from >10 to 50 mM) in the lumen, concomitantly with the increase of certain intestinal proteins involved in cell-cycle arrest. The preferential arrest of proliferation and differentiation of eukaryote cells by lactate has also been proposed (Hsiao et al., 2009). Thus, the glycolytic activity of *S. thermophilus in vivo* sustains health, through the degradation of lactose and the subsequent production of lactate.

Mucus is a major component of the intestinal barrier and is involved in the protection, defense, and homoeostasis between commensals and host (Sicard et al., 2017). A decrease in mucus thickness leads to inflammatory diseases and, hence, a bacterium which promotes mucus formation could reinforce defense (Van Herreweghen et al., 2017). The formation of mucus in the gut is a dynamic process ensured by goblet cells and driven by transcription factors, such as KLF4 (Zheng et al., 2009). The continuous production of mucus, and its thickness, composition, and penetrability are shaped by the intestinal microbiota (Wrzosek et al., 2013; Jakobsson et al., 2015; Johansson et al., 2015). For example, mucus adhesins expressed at the cell surface of *Lactobacillus reuteri* strains may exert immune regulatory effects in the gut (Bene et al., 2017; de Vos, 2017). Better adhesion of probiotics and bacteria in transition should improve persistence and favor an intimate dialog with epithelial cells. Thus, adhesion to mucus is a factor often ascribed to probiotic characteristics in the gut (FAO/WHO, 2002), but this assumption requires further studies for validation and generalization.

Although adhesion is not a necessary criterion to estimate a potential benefit, it is still an important property, together with mucin/mucin sugar degradation, for understanding the overall effect of a strain in the digestive environment. Little is known concerning the overall mucophilic potential of *S. thermophilus*, except for one recent study showing that sortase-dependent proteins are involved in its adhesion profile (Kebouchi et al., 2016). The aim of this study was to evaluate the ability of *S. thermophilus* to adhere, degrade, modify, or induce the production of mucus and/or mucins.

## Materials and Methods

### Strains and Culture Conditions

The strains *S. thermophilus* LMD-9 (ATCC BAA-491, United States) and LMG18311 (BCCM collection, Belgium) were used. Stock cultures of *S. thermophilus* LMD-9 and LMG18311 were prepared in reconstituted 10% (w/v) Nilac skim milk (NIZO, Ede, Netherlands).

Two *Lactococcus lactis* bacterial strains were used in this study: *L. lactis* subsp. *lactis* TIL448 (NCDO2110), isolated from peas, and TIL1230, a derivative of TIL448 obtained after curing plasmids by acridine orange treatment, devoid of pili and mucus-binding protein (Meyrand et al., 2013). Bacterial stock cultures were maintained at −80°C in M17 broth (Oxoid), containing 0.5% (w/v) glucose and 20% (v/v) glycerol. Bacteria were first sub-cultured overnight at 37°C (*S. thermophilus*) or 30°C (*L. lactis*) in M17-lactose [1% (w/v)] (*S. thermophilus*) or glucose [0.5% (w/v)] (*L. lactis*) medium (M17Glc). This preculture was then used to inoculate M17Glc at 37 or 30°C. Bacteria were harvested during the exponential growth phase [optical density at 600 nm (OD_600_) of approximately 3] by centrifugation (2000 × g, 20 min, room temperature) and the pellet was washed once with phosphate buffered saline (PBS).

LMD-9 *ster_0152::kana* strain (LMD-9^Kana^) was constructed as follows. The kanamycin (*kana*) cassette of the plasmid pKa (Debarbouille et al., 1990) was PCR amplified using Phusionhigh fidelity DNA polymerase (Fermentas) with AphA3F (5′CCAGCGAACCATTTGAG3′) and AphA3R (5′GTTGCGGATGTACTTCAG3′) primers. The DNA fragments flanking the *ster_0152* gene were PCR amplified using Phusion DNA polymerase, LMD-9 DNA as template, and A1 (5′CTTACCTATCACCTCAAATGGTTCGCTGGGTTT ATCTTAAAATGATTTATCGTC3′) and A3 (5′GGACAGC CGTAAACTATC3′) primers for the upstream fragment, and A2 (5′AGGGGTCCCGAGCGCCTACGAGGAATTTGTATCGA T3GGGACAGAACATTGTACC3′) and A4 (5′CATCCATTAA GACGCCCC3′) primers for the downstream fragment. The 3′ end of the generated upstream fragment contained a sequence complementary to the 5′ end of the *kana* cassette, whereas the 5′ end of the generated downstream fragment contained a sequence complementary to the 3′ end of the cassette. This allowed joining of these three fragments after TaqPhusion PCR using primers A3 and A4. After purification with a QIAquick PCR purification kit, 500 ng of the resulting fragment was further used to transform competent natural LMD-9 cells as described by Gardan et al. (2009). LMD-9^Kana^clones were selected on M17Lac plates with kanamycin (1 mg/ml) and then verified by two PCRs using oligonucleotides A1 × A3 and A2 × A4. Finally, sequencing of the flanking regions was performed to ensure that no unwanted mutations were introduced. The two strains, LMD-9^Kana^ and native LMD-9, grew similarly in M17 lactose-rich (1%, w/v) medium and milk (data not shown).

### Degradation of Mucin Sugars by *S. thermophilus*

The ability of *S. thermophilu*s LMD-9 and LMG18311 to degrade mucin sugars was evaluated by following their growth in microtiter plates with Yeast Extract medium (YE, Biokar diagnostics A1202 HA) containing 10 g/L of one of the carbon sources to be tested (galactose, mannose, fucose, *N*-acetyl glucosamine, *N*-acetyl galactosamine, or lactose) at 42°C. The growth rates are expressed as μ_Max_means ± SD (*n* = 7).

### HT29-MTX Culture

The mucus-secreting cell line HT29-MTX was obtained from Thecla Lesuffleur (INSERM Paris, France). Cells were routinely grown in Dulbecco’s modified Eagle’s minimal essential medium (DMEM) containing phenol red and 4.5 g/L glucose (Lonza, Verviers, Belgium), supplemented with 10% (v/v) heat-inactivated Fetal Bovine Serum, 1% (v/v) L-glutamine 200 mM, and 1% (v/v) penicillin-streptomycin mixture (10000 U/mL and 10000 μg/mL, respectively) (all from Lonza). Cells were seeded at a concentration of 2.5 × 10^4^ cells/cm^2^ in six-well tissue-culture plates (Thermo Fisher Scientific - Nunc A/S, Waltham, MA, United States). Two days before the experiments, antibiotics were no longer added to the cell culture medium. Experiments were carried out 20–22 days post seeding to ensure full differentiation of cells. Cells were maintained at 37°C in a humidified atmosphere with 10% CO_2_ and the culture medium was changed daily.

#### Bacterial Adhesion to HT29-MTX Cells

Adhesion experiments of *S. thermophilus* LMD-9 to the HT29-MTX cell line were performed according to the method described previously by Turpin et al. (2012), with some modifications. TIL448 and TIL1230 were used as high-adhesive and low-adhesive controls, respectively (Le et al., 2013). Bacterial cells from precultures were used to inoculate cell-culture medium with a reduced FBS concentration [2% (v/v)] at a starting OD_600__nm_ = 0.05. After an adaptation phase, bacteria were washed, suspended in fresh culture medium with 2% FBS, and diluted to obtain a bacterial-cell concentration of 3–4 × 10^7^CFU/mL. HT29-MTX cells were gently washed twice with PBS, pH 7.5 (Lonza) and 2 mL bacterial suspensions added to each well, resulting in a bacterial cell to epithelial cell ratio (MOI) of 10:1. The bacterial and epithelial cells were co-incubated for 2 h at 37°C in a humidified atmosphere with 10% CO_2_. After incubation, HT29-MTX cells were washed twice with PBS to remove unbound bacteria, scraped with 0.1% (v/v) Triton X-100 (Sigma-Aldrich, St. Louis, MO, United States), filtered five times through a 21-gauge needle, and incubated for 30 min at room temperature. The number of viable bacterial cells was determined in the cell pellet (adherent bacterial cells) and bacterial suspension after incubation in empty wells (control input) by the plating method. Results are expressed as the percentage of adherent bacterial cells relative to the number of bacteria added (control input). At least three independent experiments were performed and at least three serial dilutions in duplicate were carried out for each well.

#### Co-incubation of HT29-MTX Cells With Lactate

Cells were cultivated for 7 days and incubated with 0, 20, or 50 mM lactate (Sigma-Aldrich, St. Louis, MO, United States) for 17 h. The pH was verified and adjusted to that of normal culture medium for culture medium containing lactate. Each condition was tested in triplicate and experiments were repeated three times. Cells were scraped off and immediately used for protein extraction.

### Binding to Mucins

A solution of type III mucin from porcine stomach (PGM) (cat. no. M1778, Sigma-Aldrich, St. Louis, MO, United States) [10 mg/mL] was dissolved in phosphate-buffered saline (PBS) pH 7.5 and used to coat 96-well polystyrene microtiter plates (Thermo Fisher Scientific - Nunc A/S). Adhesion of *S. thermophilus* LMD-9 and LMG18311 to PGM-coated polystyrene plates was tested using the technique described for the *L. lactis* IBB477 strain (Radziwill-Bienkowska et al., 2016). TIL448 and TIL1230 were used as high-adhesive and low-adhesive controls, respectively (Le et al., 2013). Briefly, bacterial suspensions (OD_600__nm_ = 1) were incubated in coated plates for 3 h at room temperature, unbound bacteria washed away, and adherent bacteria stained with crystal violet before solubilization in acetic acid. Adhesion is expressed as the optical density (OD_583__nm_) of stained cells. Bacterial adhesion was determined in three independent experiments and results are presented as the means ± SD. The average value of at least six measurements was calculated after rejecting extreme results.

### Adhesion to Purified Human Mucins

#### Approval and Accordance for Human Samples

Colorectal tissues from healthy individuals arised from patients with diverticulosis. The use of human tissues for this study was approved by the local hospital ethics committee and French Ministry of Higher Education and Research (DC-2008-242). All subjects gave written informed consent in accordance with the Declaration of Helsinki.

The procedure was adapted from that of Da Silva et al. (2015) to evaluate the binding of *S. thermophilus* LMD-9 to human colonic mucin. TIL448 and TIL1230 were used as high-adhesive and low-adhesive controls, respectively (Le et al., 2013). In brief, mucin was scraped and purified from human biopsies and spotted (10 μg) on dry nitrocellulose membranes. PGM was used as control. Bacteria (10^9^ CFU/mL in phosphate-buffered saline) were stained with DAPI for 15 min at room temperature in the dark. Labeled bacteria were added to the membrane in blocking buffer. After incubation for 1 h at room temperature in the dark, the fluorescence of adherent bacteria was detected by a ChemiGenius 2 imaging system (Syngene).

### Animals and Experimental Design

#### Approval and Accordance

All procedures for gnotobiotic rats were carried out according to European guidelines for the care and use of laboratory animals with permission from the French Veterinary Services dedicated to M. T (78-123) and as previously described by Rul et al. (2011).

All mice experiments were approved by the local Animal Ethics Review Committee of the Faculty/Paris 7 University with permission dedicated to the unit of EOD [EU0543 - Fac Méd X. Bichat – Animal. Centr. - APAFiS - Autor. APAFiS #6424].

Ino-LMD9: All mono-colonized rats were previously described by Rul et al. (2011). Briefly, 2-month-old germ free (GF) rats (male, Fisher 344) were inoculated by a single gavage with 1 mL 5 × 10^8^ CFU/mL *S. thermophilus* LMD-9 (Ino-LMD9, *n* = 6). As a control, GF rats (*n* = 5) were also inoculated with 1 mL sterile Nilac milk (without bacteria). All gnotobiotic rats for which the drinking water was supplemented with lactose (4.5%wt/vol) are designated as ^+lac^ (GF^+lac^, *n* = 6 and Ino-LMD9^+lac^, *n* = 6). All rats were euthanized 30 days after gavage to recover biological samples.

Ino-LMD9^Kana^ mice: Male C57BL/6J mice were obtained from Charles River Laboratories. After receipt, mice were acclimated for 1 week and then randomized into control (*n* = 5) and treated (*n* = 5) groups. Mice were housed in a conventional animal facility under a 12:12-h light/dark cycle. Food and tap water were provided *ad libitum*. Five individuals per cage were housed in the same room. At the age of 4 weeks, mice were inoculated by oral gavage (10^8^ CFU/mL in 150 μL/mouse), five times a week (from Monday to Friday), at 10 a.m. for 3 weeks, either with *S. thermophilus* LMD9^Kana^ (Ino-LMD9^Kana^, *n* = 5) or Nilac skim milk (Ctrl, without bacteria) (*n* = 5). Stool samples were collected two times a week at 4 p.m. All mice were euthanized at the age of 7 weeks by cervical dislocation.

### Histology and Immunohistochemistry Assays

Flushed colons of rats and mice were opened longitudinally and cut into 2-cm sections. The samples were fixed in 4% paraformaldehyde (4 h, room temperature), dehydrated, and embedded in paraffin, according to standard histological protocols. Four-micrometer thick sections of rat distal colon (proximal, median, and distal for mice) were mounted on SuperFrost^®^ Plus slides (Thermo Fisher, Waltham, MA, United States). Paraffin-embedded sections were deparaffinized and stained with Hematoxylin and Alcian blue (AB) solution pH 2.5 to count the number of total and goblet cells per crypt, respectively. MUC2 immunostaining was performed for rats using the EnVision + System-HRP (Dako-Cytomation, Trappes, France) and anti-MUC2 antibody (1:5000; sc-15334, Santa Cruz Biotechnology, Heidelberg, Germany), as previously described (Tomas et al., 2013). Only U-shaped longitudinally cut crypts with open lumina were counted. The reported results are the means obtained by analysis of at least 10 crypts per site using Nanozoomer Digital Pathology view software (Hamamatsu Photonics, Hamamatsu, Japan).

### Western Blot Analysis

Colonic epithelial cells were isolated from the whole colon of rats and mice according to the method of Cherbuy et al. (1995). The cell pellet from whole colon or HT29-MTX cell cultures was immediately used for protein extraction (Cherbuy et al., 2010) and Lowry’s procedure was used for protein assays. Western blot analysis was performed using 12% SDS-PAGE and anti-KLF4 (1:500; IMG-6081A, Imgenex, San Diego, CA, United States), with an appropriate peroxidase-conjugated secondary antibody (Jackson ImmunoResearch laboratories, West Grove, PA, United States). For each western blot, protein loads were determined using anti-cullin1 (1:200; sc-17775, Santa Cruz Biotechnology), or anti-GAPDH (1:1000; 905-734-100, Stressgen) antibodies. Signals detected on autoradiographic films were quantified by scanning densitometry using a Biovision 1000 and Bio-1D software (Vilber Lourmat, France).

### Total RNA Extraction and Real-Time Quantitative PCR Analysis

Total RNA was extracted from colonic epithelial cells isolated from rat tissues by the guanidinium thiocyanate method (Chomczynski and Sacchi, 1987). RNA concentration and purity were determined by absorbance using a Nanodrop ND-1000 (Thermo Fisher Scientific, Illkirch, France) and the RNA Integrity Number (RIN) determined using an RNA 6000 Nano LabChip^®^ kit (Agilent Technologies, Santa Clara, CA, United States) and an Agilent 2100 bioanalyzer at the ICE platform (INRA, Jouy-en-Josas, France). All RNA had a RIN between 8 and 10, indicating that the RNA of all samples was of high quality. Reverse transcription was performed with 7 μg RNA of each sample using the High-Capacity cDNA Archive Kit (Applied Biosystems by Life Technologies SAS, Saint Aubin, France) according to the manufacturer’s instructions. No inhibition was detected for any sample using the TaqMan^®^ Exogenous Internal Positive Control (Applied Biosystems). The cDNA products were analyzed in triplicate by RT-qPCR with an ABI PRISM 7000 Sequence Detection System and 7000 system software, version 1.2.3 (Applied Biosystems). Muc2 mRNA was analyzed using TaqMan Gene Expression Assays (Rn01498197_m1, Applied Biosystems. 18S rRNA (Hs99999901_s1) was used as a reference. Results obtained were normalized to the value for 18S rRNA and compared with the mean target gene expression in GF rats as the calibrator sample. The following formula was used: fold change = 2^−ΔΔCt^, where ΔΔCt threshold cycle (Ct) equals (target Ct – reference Ct) of the sample minus (target Ct – reference Ct) of the calibrator.

### Metabolite Analysis

Acetate, propionate, butyrate, valerate, isobutyrate, and isovalerate concentrations were determined in cecum contents of mice after water extraction of acidified samples by gas liquid chromatography (Nelson 1020, Perkin-Elmer, St Quentin en Yvelines, France), as previously described (Lan et al., 2008). Lactate measurements were performed from the cecum content of rats and mice, as previously described (Ben-Yahia et al., 2012). The metabolite concentrations are expressed in mM.

### Analysis of Mucin *O*-glycosylation in Mice

Distal colonic mucosa was scraped and the mucins solubilized and purified by isopycnic density-gradient centrifugation (Beckman Coulter LE80K ultracentrifuge; 70.1 Ti rotor, 308, 500 × g at 15°C for 72 h) (Rossez et al., 2012). The mucin-containing fractions were pooled, dialyzed into water, lyophilized, and further submitted to β-elimination under reductive conditions [0.1 M NaOH, 1 M KBH4 for 24 h at 45°C]. Permethylation of the mixture of oligosaccharide alditols was carried out using the sodium hydroxide procedure. After derivation, the reaction products were dissolved in 200 μL methanol and further purified on a C18 Sep-Pa cartridge (Waters, Milford, MA, United States). Permethylated oligosaccharides were analyzed by MALDI-TOF mass spectrometry in positive ion reflective mode as [M+Na]^+^. The relative percent of each oligosaccharide was calculated based on the integration of peaks of MS spectra.

### Statistical Analysis

Statistical analysis was performed using GraphPad Prism software (GraphPad Software Inc., La Jolla, CA, United States). For *in vitro* binding experiments, data are reported as the means ± standard errors of the means (SEM) and one-way analysis of variance (ANOVA), followed by Tukey’s Multiple Comparison test, was performed to compare data groups. Asterisks indicate values significantly different from the high-adhesive *L. lactis* strain or from low-adhesive *L. lactis* stain (^∗∗∗^*p* < 0.001 and ^∗∗^*p* < 0.01). For animal experiments, data are reported as the means ± standard errors of the means (SEM) and *t*-test or ANOVA, followed by Tukey’s Student range test, were performed to compare the data between different batches of animals (^∗^*p* < 0.05).

## Results

### *In Vitro* Mucus-Adhesion and Degradation of Mucin Sugars by *S. thermophilus*

**FIGURE 1:**
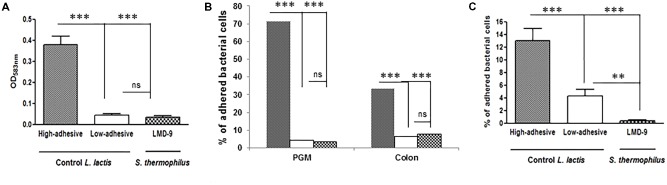
*Invitro* adhesion of *S. thermophilus* LMD-9(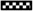) *vs.* the high-adhesive *L. lactis*TIL448 (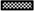) and low-adhesive *L. lactis*TIL1230 (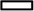). **(A)** PGM-coated polystyrene microplates: adhesion is expressed as optical density (OD_583__nm_) of stained cells. **(B)** Human colonic mucin spotted on nitrocellulose membranes: adhesion is expressed as the percentage of adherent bacterial cells relative to the number of bacteria added. **(C)** Mucus-secreting HT29-MTX cells: adhesion is expressed as the percentage of adherent bacterial cells relative to the number of bacteria added. The means ± SEM from three independent experiments are shown (^∗∗∗^*p* < 0.001, ^∗∗^*p* < 0.01, ns, not significant).

We established various models to assess the ability of *S. thermophilus* to bind mucus and mucins. We used two types of purified mucins: commercial pig gastric mucin (PGM) and colonic mucin isolated from human biopsies. The LMD-9 strain was co-incubated with PGM coated on polystyrene microplates (**Figure 1A**) or human mucin *vs.* PGM spotted on nitrocellulose membranes (**Figure 1B**). The TIL448 *L. lactis* strain, known to be highly adhesive (Le et al., 2013; Meyrand et al., 2013), was used as a positive control and indeed bound to PGM. In contrast, *S. thermophilus* LMD-9 bound as poorly as our negative *L. lactis* control, TIL1230 (**Figures 1A,B**). The adhesive profile of *L. lactis* TIL448 was also high for human samples, whereas that of *S. thermophilus* LMD-9 was low, identical to that of TIL1230 (**Figure 1B**). We also used intestinal HT29-MTX cells to mimic the continuous and dynamic production of mucus (**Figure 1C**). Binding of the *S. thermophilus* LMD-9 strain to HT29-MTX cells was also low. Thus, *S. thermophilus* LMD-9 displayed poor binding to mucus and mucins relative to that of the high-adhesive *L. lactis* TIL448 strain, regardless of the model tested (**Figure 1**). The low ability of *S. thermophilus* to bind mucin was confirmed with the other strain tested, LMG18311 (data not shown). We then evaluated the ability of *S. thermophilus* to use the sugars forming the mucin glycans as their sole carbon source. The growth of *S. thermophilus* was characterized in microplates with culture medium supplemented with galactose, mannose, fucose, *N*-acetyl glucosamine, *N*-acetyl galactosamine, or lactose, which is the preferred substrate of *S. thermophilus*. The maximal growth rate (μ_max_) of LMD-9 was 0.27 ± 0.08 h^−1^ in the presence of lactose, whereas this strain did not grow in any of the sugars forming mucins as their sole carbon source. We obtained similar results with the LMG18311 strain, with a μ_max_ reaching 0.30 ± 0.05 h^−1^ in the presence of lactose and no growth in the presence of the mucin sugars. Overall, these data show that two strains of *S. thermophilus* (LMD-9 and LMG18311) adhere poorly to mucus and mucins *in vitro*, and are unable to grow *in vitro* with mucin sugars as the sole carbon source.

### Mucus-Induction by *S. thermophilus* in a Simplified *in Vivo* Gnotobiotic Model

**FIGURE 2:**
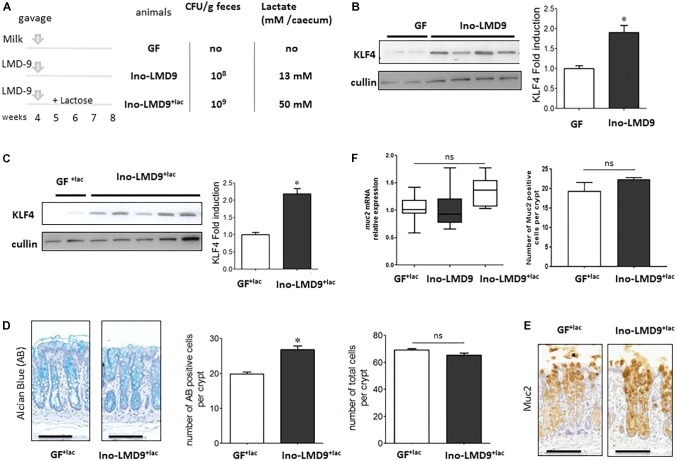
Characterization of the colonic epithelial response in *S. thermophilus*LMD-9 mono-colonized rats. Germfree (GF) rats were inoculated with sterile milk (GF, *n* = 5) or with 5 × 10^8^ CFU of a culture of *S. thermophilus* LMD-9 (Ino-LMD9, *n* = 6) and euthanized 30 days after inoculation. Similar groups GF^+lac^ (*n* = 6) and Ino-LMD9^+lac^ (*n* = 6) were given tap water containing 4.5% (w/v) lactose **(A)** Experimental design: establishment of *S. thermophilus* LMD-9 (with or without lactose) in the gastrointestinal tract was monitored by enumeration of the bacterial counts in the feces. The lactate concentration was measured in the cecum. **(B)** Representative western blot and densitometric analyses of KLF4 protein in GF and Ino-LMD9 samples. **(C)** Representative western blot and densitometric analyses of KLF4 protein in GF^+lac^ and Ino-LMD9^+lac^ samples. **(D)** Representative images, counts of goblet cells stained with Alcian Blue (indicated as AB) and counts of total number of cells per crypt stained with Hematoxylin from in GF^+lac^ and Ino-LMD9^+lac^ samples; scale bar: 50 μm. **(E)** Representative images, Muc2 immunostaining in GF^+lac^ and Ino-LMD9^+lac^ rats; scale bar: 50 μm. **(F)** *muc2* mRNA expression level in GF^+lac^, Ino-LMD9 and Ino-LMD9^+lac^ rats, and counts of the number of muc2 positive cells per crypt GF^+lac^ and Ino-LMD9^+lac^ rats. The asterisk indicates a statistical difference relative to GF rats (*p* < 0.05); ns: not significant.

**FIGURE 3:**
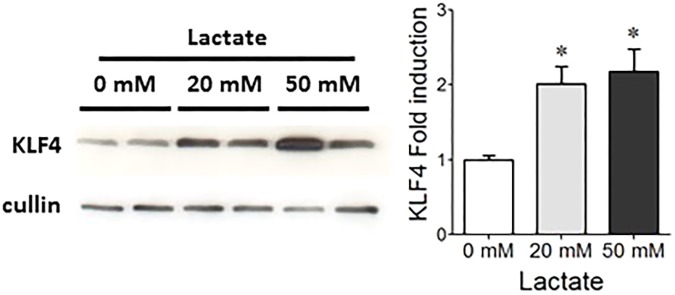
Effect of lactate on KLF4 protein levels in HT29-MTX cells. Representative western blot and densitometric analyses of KLF4 protein in HT29-MTX cells incubated with 0, 20, or 50 mM lactate. The means ± SEM from three independent experiments are shown (^∗^*p* < 0.05).

We tested the ability of *S. thermophilus* to modulate the mucus pathway of intestinal epithelial cells by studying mono-colonized adult rats, harboring LMD-9 as the sole bacterium in their digestive tract. Ino-LMD9 rats, which we previously described (Rul et al., 2011), were colonized by up to 10^8^CFU/g feces and accumulated 13 mM of lactate in the cecum (**Figure 2A**). When lactose (4.5% w/v) was added in drinking water (Thomas et al., 2011), the level of colonization was 10^9^ CFU/g feces and lactate accumulated up to 50 mM in mono-colonized Ino-LMD9^+lac^rats (**Figure 2A**). The presence of *S. thermophilus* in the digestive tract induced the amount of KLF4 protein in epithelial cells of the colon in two groups without and with lactose (**Figures 2B,C**). The level of induction is 1.9 (±0.6) in Ino-LMD9 and 2.1(±0.46) in Ino-LMD9^+lac^ in comparison with their respective germ free counterparts (*p* < 0.05) and the two groups of mono-associated rats (with and without lactose) displayed similar amount of KLF4 protein. There was also a comparable induction of KLF4 in mono-colonized rats carrying the LMG18311 strain (Supplemental **Figure 1**). Goblet cells stained by Alcian Blue were more abundant in Ino-LMD9^+lac^ than with GF^+lac^ rats (**Figure 2D**), whereas the number of total cells per crypt was the same. The production of Muc2 protein, the main mucin secreted in the colon, seems to be higher in the Ino-LMD9^+lac^ rats than in GF (**Figure 2E**), but the mRNA level and the number of Muc2 positive cells were unchanged after colonization of the colon by *S. thermophilus* LMD9^+lac^ (**Figure 2F**). Overall, these data show that two strains of *S. thermophilus*, LMD9 and LMG18311, induced KLF4 protein in mono-colonized rats in the presence or not of a continuous lactose supply, concomitantly with the accumulation of high amount of lactate (from 13 to 50 mM) in their digestive tract. We then evaluated the effect of lactate (20 and 50 mM) on KLF4 protein production by the mucus-producing HT29-MTX cell line (**Figure 3**). Lactate significantly increased the amount of KLF4 and the level of induction was equivalent regardless of the concentration tested (20 or 50 mM *in vitro*). Overall, *S. thermophilus* can induce KLF4 protein and the number of Alcian-Blue positive goblet cells when it is the only bacterium in the digestive tract of rats. Based on our observations in HT29-MTX cell line, we can suppose that lactate may be involved in the induction of KLF4.

### Mucus-Related Properties of *Streptococcus thermophilus* in Mice Harboring a Complex Microbiota

**FIGURE 4:**
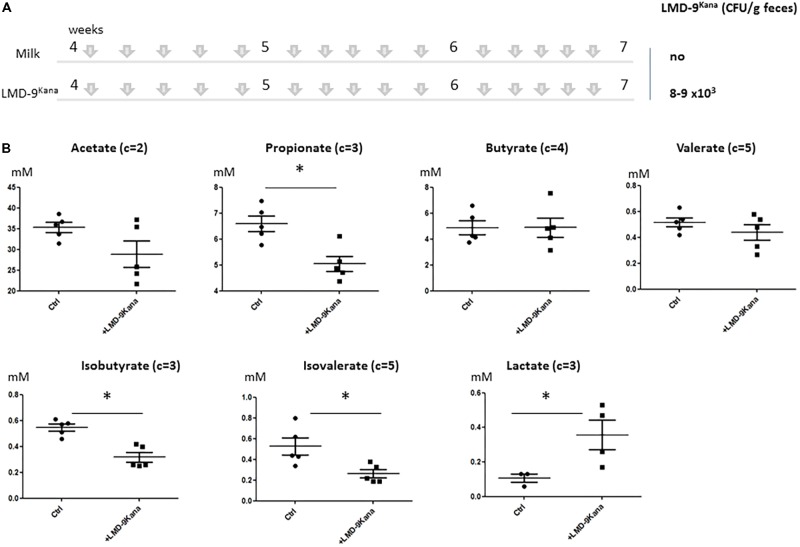
Impact of *S. thermophilus* LMD-9 on the fermentative profile of the gut microbiota in conventional mice. C57BL/6J mice were inoculated by oral gavage (150 μL of 10^8^ CFU/mL/mouse), five times a week, for 3 weeks, with either *S. thermophilus* LMD9^Kana^ (Ino-LMD9^Kana^, *n* = 5) or sterile milk (Ctrl, *n* = 5). **(A)** Experimental design; establishment of *S. thermophilus*LMD-9 in the gastrointestinal tract was monitored by enumeration of the bacterial counts in the feces. **(B)** Cecal composition of acetate, propionate, butyrate, valerate, isobutyrate, isovalerate, and lactate. The number of carbon parts per substrate is indicated for each metabolite. The asterisk indicates a statistical difference compared to Ctrl mice (*p* < 0.05).

**FIGURE 5:**
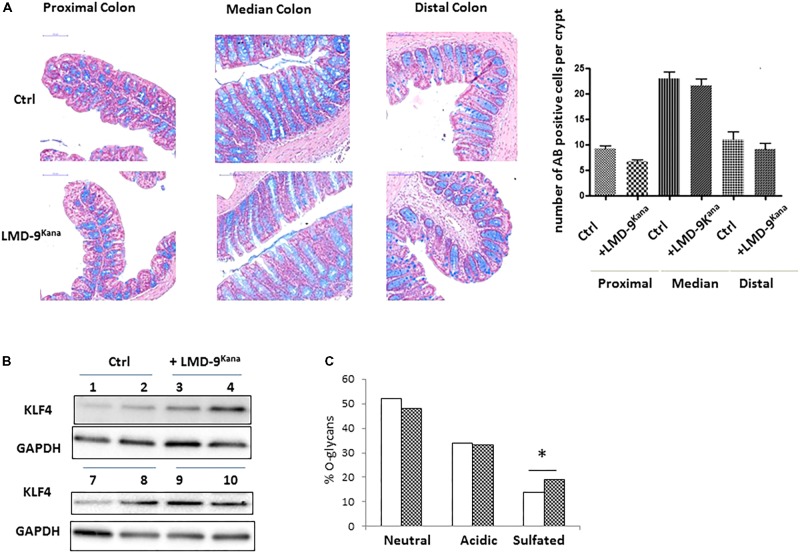
Characterization of the colonic epithelial response to *S. thermophilus* LMD-9 in conventional mice. **(A)** Representative pictures and counts of goblet cells stained with Alcian Blue (indicated as AB) in proximal, median, and distal colon of Ctrl (*n* = 5) and Ino-LMD9^Kana^ (*n* = 5) samples; scale bar: 100 μm. **(B)** Representative western blot of KLF4 protein (involved in the mucus pathway) in Ctrl and Ino-LMD9^Kana^ samples. **(C)** Proportion of neutral, acidic, and sulfated *O*-glycans in Ctrl (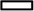, *n* = 5) and Ino-LMD9^Kana^ (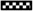, *n* = 5) samples. The asterisk indicates a statistical difference relative to Ctrl mice (*p* < 0.05).

The use of mono-colonized rodents allowed us to assess several mucus-related functions in an over-simplified digestive system. We next characterized the effect of *S. thermophilus* in mice harboring a complex microbiota by constructing an LMD-9 mutant strain resistant to kanamycin (LMD-9^Kana^), enabling the evaluation of the colonization level of *S. thermophilus* in a complex bacterial community. The adult mice received either milk fermented with LMD-9^Kana^ or only milk (Ctrl) five times per week for 3 weeks (**Figure 4A**). We detected 8–9 10^3^ CFU/g feces of LMD-9^Kana^ (**Figure 4A**), showing that live bacteria transited through the digestive tract, whereas no LMD-9^Kana^ was present in the Ctrl group, as expected. We assessed the fermentative profile of the microbial community by measuring the levels of short chain fatty acids and lactate in the cecum (**Figure 4B**). Mice receiving LMD-9^Kana^ had significant levels of lactate (up to 0.5 mM) in the cecum and lower levels of propionate, isobutyrate, and isovalerate than the Ctrl group (*p* < 0.05). Thus, the transiting of *S. thermophilus* as sub-dominant bacteria, may orientate the activity of the resident and abundant microbiota. The number of goblet cells and KLF4 protein levels in the colon were not different in the mice that received LMD-9^Kana^ than in Ctrl mice (**Figures 5A,B**). Probiotic or commensal bacteria are known to modulate the composition and physical properties of mucus through changes in mucin *O*-glycans (Wrzosek et al., 2013; Da Silva et al., 2014). We thus profiled the *O*-glycosylation pattern of colonic mucins. The number of sulfated mucins was significantly higher in mice receiving LMD-9^Kana^ than in Ctrl mice (**Figure 5C**). Thus, transit of *S. thermophilus* as a sub-dominant bacterium through the microbiota resulted in modification of some bacterial metabolite levels, with lactate levels, no induction of the mucus pathway, and a moderate induction of sulfated mucin.

## Discussion

In this study, we characterized the overall interactions between the food-borne bacterium *S. thermophilus* and mucus, using a battery of tests on *in vitro* and *in vivo* models. Two strains of *S. thermophilus*, LMD-9 and LMG18311, poorly adhered to mucus and mucins (**Figure 1**) and were unable to degrade various sugars used in mucin synthesis. *S. thermophilus* is thus a poor adhesive bacterium relative to other well-described mucus-adhesive lactic-acid bacteria, such as *Lactobacillus reuteri* and *Lactobacillus plantarum* (Mackenzie et al., 2010; Turpin et al., 2012). Adhesion is clearly not the most determinant trait of *S. thermophilus*.

We have administrated *S. thermophilus* in recipient germ free rats and in conventional mice to characterize its effect on mucus pathway in two complementary animal models. The rats received 1ml of pure *S. thermophilus* culture (5^∗^10^8^ CFU/ml), equivalent to the amount of *S. thermophilus* in ≈50 g of yogurt. In mice, we have administrated 5 times per week for 3 weeks 0.15 ml of pure *S. thermophilus* culture of 10^8^ CFU/ml, equivalent to ≈1.5 g of yogurt per gavage.

The number of Alcian-Blue positive goblet cells and the level of KLF4 protein were induced in the simplified Ino-LMD9 model, in which *S. thermophilus* is the only bacterial species in the digestive tract (**Figure 2**). Mucus reinforcement is one of the mechanisms responsible for the beneficial effects of several lactic-acid bacteria. For example, *Lactobacillus rhamnosus* GG up-regulates mucin gene expression and mucus production in intestinal epithelial cell lines through two actors: p40-derived soluble protein of the bacteria and the EGF receptor of host cells (Mattar et al., 2002; Wang et al., 2014). Adhesion to mucus has also, often been proposed to favor colonization and thus exchanges between bacteria and host. But, it has been recently shown that a key factor involved in *in vitro* adhesion of the *Lactococcus lactis* IBB477 strain did not confer a selective advantage in the intestinal tract of conventional mice (Radziwill-Bienkowska et al., 2017). The ability of lactic-acid bacteria to colonize the gut in animal models harboring a simplified microbiota is not determined by their ability to bind mucus (Turpin et al., 2013). The assumption that high adhesion confers a selective advantage in the gut and provides health benefits is thus not implicit. The transit time and metabolic activity in the gut result from multiple complex parameters, including resistance to bile salts and acidity, endogenous immune defense, microbial competitive advantage, and the availability of energy sources. The activity and metabolism of commensals that are highly specialized in the consumption of mucins or complex polysaccharides are associated with the amount of fiber in the diet (Martens et al., 2011). Genomic analysis suggests that LMD-9 lacks polysaccharide-degrading loci. We have shown that lactose boosts the metabolic activity and colonization of *S. thermophilus* in a simplified model, Ino-LMD9 (Thomas et al., 2011; Ben-Yahia et al., 2012), which correlated with its well-known efficient lactose permease and high β-galactosidase activities (Hols et al., 2005). Thus, the amount of mono and di-saccharides in the diet should determine the activity of *S. thermophilus* in the gut, especially in the upper part of the gut, in which high levels of free simple sugars are present.

Our *in vitro* co-incubation experiments (**Figure 3**) suggest that lactate produced by *S. thermophilus* in gnotobiotic model may sustain at least in part the stimulation of KLF4, involved in differentiation of secretive cells. This is consistent with the stimulation of cell-cycle arrest proteins and the anti-proliferative effect described for lactate *in vivo* and *in vitro* (Rul et al., 2011; Matsuki et al., 2013). However, we have previously reported that lactate accumulation produced by a mixture of three lactobacilli has no effect on mucus production in gnotobiotic rodents (Turpin et al., 2013). Thus, it is likely that the induction of the mucus pathway requires lactate and additional factors brought by *S. thermophilus* during its transit through the gut. It is possible that bioactive muco-inducer peptides may be produced by *S. thermophilus* in the gut as in yogurt. Indeed, Plaisancie et al. (2013, 2015) and Bruno et al. (2017) have shown that lactic acid bacteria of yogurt form small and functional peptides by hydrolyzing milk caseins, and some, such as peptide β-CN(94-123), significantly induce mucus production *in vitro* and *in vivo*. It may be informative to purify small peptides produced by *S. thermophilus* in the lumen that may act in synergy with lactate to induce mucus and reinforce the innate barrier and assess their function in relevant models.

We no longer observed induction of the mucus pathway in conventional mice harboring an endogenous microbiota in which *S. thermophilus* was sub-dominant (**Figure 4**). Regular gavage of these mice with *S. thermophilus* LMD-9^Kana^ did not alter the number of goblet cells nor KLF4 protein levels in the colon (**Figure 5**) or ileum (data not shown). The *O*-glycosylation pattern of mucins plays an important role in intestinal homeostasis (Bergstrom and Xia, 2013) and we found that administration of *S. thermophilus* LMD-9^Kana^ induced subtle changes in mucin *O*-glycan content in the colon. Indeed, we detected more sulfated structures in treated mice than in control animals. Of note, sulfated mucin-type *O*-glycans can exert a protective effect against colonic inflammation, as shown in chemically induced experimental colitis in mice (Tobisawa et al., 2010). It may thus be informative to evaluate whether and how *S. thermophilus* contributes to mucus maintenance in intestinal diseases or pathophysiological conditions in which the mucus barrier is altered. Indeed, it has been observed that the supplementation with *Lactobacillus plantarum* WCFS1 has no effect on mucus in conventional mice, but prevents the deterioration of the colonic mucus layer associated with aging (van Beek et al., 2016).

Supplementation of conventional mice with *S. thermophilus* led to slight modifications of the levels of several short chain fatty acids (propionate, isobutyrate and isovalerate) and lactate was detectable in the cecum at higher levels than in non-treated animals, although the difference was small. Under normal conditions, lactate does not accumulate in the intestinal lumen of adults, because other bacteria consume it (Belenguer et al., 2011; Munoz-Tamayo et al., 2011). Higher lactate concentrations are found in exclusively breast-fed infants (Bridgman et al., 2017). In the gut, lactate has anti-microbial activity by acidifying the luminal fluid and through indirect mechanisms that influence the expression of colonization factors necessary for the commensalism of pathogens. Notably, lactate lowers the transcription of genes required for *in vivo* growth and catabolism of *Campylobacter jejuni* (Luethy et al., 2017). A lactate-rich environment inside the gut may also promote tolerance to commensal bacteria (Angelin et al., 2017) and attenuate pro-inflammatory pathways (Iraporda et al., 2015; Iraporda et al., 2016). Therefore, lactate possesses a wide panel of beneficial activities inside the gut that may feed microbial communities, fight pathogens, alleviate inflammatory processes, and strengthen the intestinal barrier through mucus production. We suggest that lactate production by *S. thermophilus* in the digestive tract may contribute to the microbiota trophic chain and stimulate the mucus pathway and mucus formation under specific conditions (old age, inflammation, etc.).

Overall, the lactose-degrading probiotic *S. thermophilus* adheres poorly to mucus and poorly degrades mucin sugars *in vitro*. We propose that lactate, produced in the gut by *S. thermophilus* may potentially induce pathways involved in mucus induction, as revealed using gnotobiotic rodents to study the novel functions of transient food-borne bacteria. This suggests that the consumption of fermented dairy products may reinforce mucosal integrity of the intestine. Although we did not observe mucus induction in a more sophisticated physiological context with a complex microbiota, we believe that this newly revealed *S. thermophilus*-driven function may be protective under certain conditions in which the mucus barrier is altered.

## Author Contributions

MC-B, FR, EO-D, MT, and MM-B designed the project, obtained the financial support, and supervised the experiments. NF, LW, JR-B, BR-D, M-PD, M-LN, VL, VR, CC, M-LD-M, RL, and CR-M produced and/or analyzed the results. MT and MM-B wrote the article. All the authors have read and approved the last version of manuscript.

## Conflict of Interest Statement

The authors declare that the research was conducted in the absence of any commercial or financial relationships that could be construed as a potential conflict of interest.
