# Supplementary figures and images for: Characterization of Mucus-Related Properties of Streptococcus thermophilus: From Adhesion to Induction
Source: Front Physiol. 2018 Jul 24;9:980. doi: 10.3389/fphys.2018.00980 (PMC6067005; doi:10.3389/fphys.2018.00980)

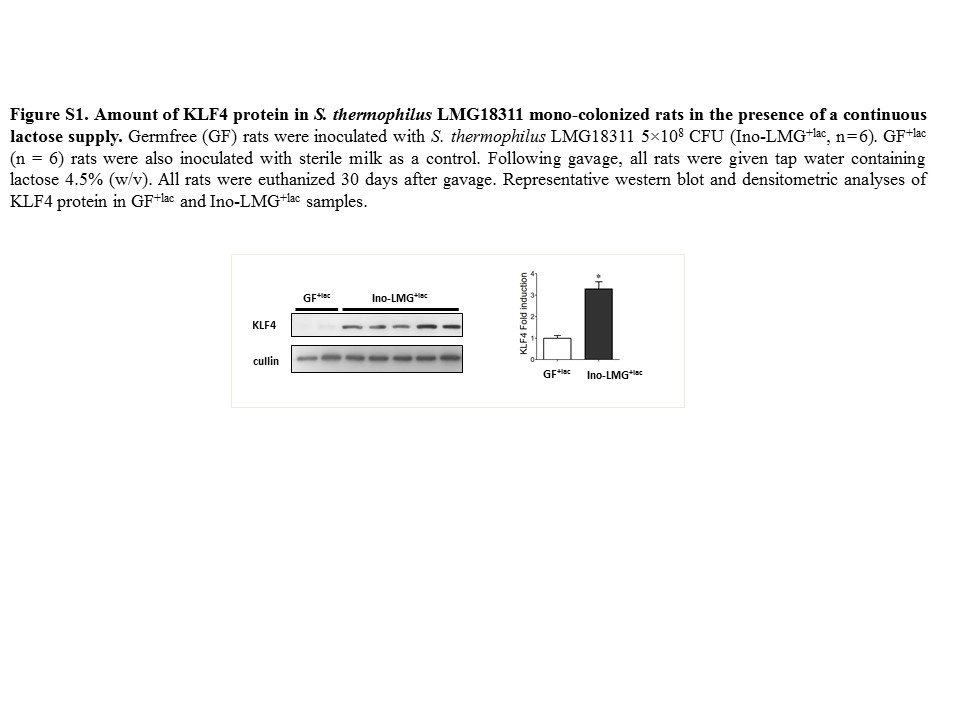

Supplement: Supplementary file 1 [file Image_1.JPEG]
